# A 12-year study evaluating the outcomes and predictors of mortality in critically ill cancer patients admitted with septic shock

**DOI:** 10.1186/s12885-021-08452-w

**Published:** 2021-06-16

**Authors:** Wedad B. Awad, Lama Nazer, Salam Elfarr, Maha Abdullah, Feras Hawari

**Affiliations:** 1 Department of Pharmacy, King Hussein Cancer Center, P.O. Box 1269, Al-Jubeiha, Amman, 11941 Jordan; 2 Department of Medicine, King Hussein Cancer Center, Amman, Jordan

**Keywords:** Sepsis, Septic shock, Cancer, Oncology, Critical care outcomes, Intensive care units

## Abstract

**Background:**

Though sepsis is common in patients with cancer, there are limited studies that evaluated sepsis and septic shock in this patient population. The objective of this study was to evaluate the outcomes and to identify predictors of mortality in cancer patients admitted to the intensive care unit (ICU) with septic shock.

**Methods:**

This was a retrospective study conducted at a medical-surgical oncologic ICU of a comprehensive cancer center. Adult cancer patients admitted with septic shock between January 1, 2008 and December 31, 2019 were enrolled. Septic shock was defined as an ICU admission diagnosis of sepsis that required initiating vasopressors within 24 h of admission. Patient baseline characteristics, ICU length of stay and ICU and hospital mortality were recorded. Univariate analysis and logistic regression were performed to identify predictors associated with ICU and hospital mortality.

**Results:**

During the study period, 1408 patients met the inclusion criteria. The mean age was 56.8 ± 16.1 (SD) years and mean Acute Physiology and Chronic Health Evaluation (APACHE) II was 23.0 ± 7.91 (SD). Among the enrolled patients, 67.8% had solid tumors while the remaining had hematological malignancies. Neutropenia and thrombocytopenia were reported in 19.3 and 39.5% of the patients, respectively, and mechanical ventilation was required for 42% of the patients. Positive cultures were reported in 836 (59.4%) patients, most commonly blood (33%) and respiratory (26.6%). Upon admission, about half the patients had acute kidney injury, while elevated total bilirubin and lactic acid levels were reported in 13.8 and 65.2% of the patients, respectively. The median ICU length of stay was 4 days (IQR 3–8), and ICU and hospital mortality were reported in 688 (48.9%) and 914 (64.9%) patients, respectively. Mechanical ventilation, APACHE II, thrombocytopenia, positive cultures, elevated bilirubin and lactic acid levels were significantly associated with both ICU and hospital mortality.

**Conclusions:**

In a relatively large cohort of patients with solid and hematological malignancies admitted to the ICU with septic shock, hospital mortality was reported in about two-third of the patients. Mechanical ventilation, APACHE II, thrombocytopenia, positive cultures, elevated bilirubin and lactic acid levels were significant predictors of mortality.

## Background

Sepsis is a common complication in cancer patients due to the immune-suppression associated with the underlying malignancy and the various cancer-related therapies [1]. In a large cohort of over 1 million sepsis hospitalizations in the US, 1 in 5 admissions were cancer-related [2]. Furthermore, in critically ill patients with cancer, sepsis was among the most common admission diagnosis to the intensive care units (ICUs) [3–5]. Though the outcomes of critically ill cancer patients have improved over the years, studies continue to report a higher mortality in cancer patients with sepsis, compared to non-cancer patients [2, 6–11].

Septic shock is a subset of sepsis which involves the initiation of vasopressors to maintain adequate arterial blood pressure, despite fluid resuscitation. The underlying circulatory, metabolic, and cellular abnormalities in septic shock are associated with high hospital mortality, approaching 40–60% [12].

Though several studies have evaluated sepsis in cancer patients, most did not specifically evaluate the subset of patients with septic shock. In addition, most of the studies included relatively small sample sizes, were conducted over a short period of time, or included a specific subset of cancer patients [4, 5, 13–21]. Therefore, we conducted this study to evaluate the outcomes of cancer patients admitted to the ICU with septic shock and to identify predictors of mortality in this patient population.

## Methods

This was a retrospective study conducted at King Hussein Cancer Center, a 370-bed comprehensive cancer teaching hospital located in Amman, Jordan. The hospital has two medical-surgical oncologic critical care units that serve around 800 patients per year, who are admitted with cancer and non-cancer related critical illnesses. The ICU has a closed-unit model, with the most common admission diagnosis being respiratory failure and sepsis and an overall ICU mortality of 35% [3]. The study was approved by the institutional review board, with a waiver of informed consent.

The study included adult patients (≥ 18 years) with solid and hematologic malignancies who were admitted to the ICU with septic shock between January 2008 and December 2019. Septic shock was defined as having an admission diagnosis of sepsis along with the initiation of vasopressors within 24 h of ICU admission.

The pharmacy medication database was used to identify patients who received norepinephrine during the study period. Norepinephrine was the vasopressor we searched for since it is the first line vasopressor used in our patients with septic shock, as per the recommendations of the Surviving Sepsis Campaign guidelines [22]. The patient medical records were reviewed to determine the admission diagnosis of the patients. Patients who received norepinephrine within the first 24 h of their ICU admission and had an admission diagnosis of sepsis were included. Patients who were started on norepinephrine after 24 h of their ICU admission and those who were prescribed norepinephrine but the infusion was not initiated, based on the medical notes, were excluded.

Using the electronic patient medical records, the following patient characteristics were recorded: patient demographics, Acute Physiology and Chronic Health Evaluation (APACHE) II score, type of malignancy, chemotherapy received within 1 month of ICU admission, Surveillance Epidemiology and End Results (SEER) stage, smoking history, absolute neutrophil count (ANC) and platelet counts upon ICU admission, the presence of positive cultures, type of microorganisms, ICU length of stay and ICU and hospital mortality. For organ dysfunction; mechanical ventilation, serum creatinine, total bilirubin and lactic acid levels upon ICU admission were recorded. Neutropenia and thrombocytopenia were defined as having ANC of less than 1000 and platelet count of less than 100*10^3/ul, respectively. Acute kidney injury was defined as an increase in serum creatinine by 0.3 mg/dl or more within 48 h. Total bilirubin and lactic acid levels were considered elevated if the level upon ICU admission was equal to or higher than 2.5 mg/dl and 2 mmol/l, respectively. The data was entered into a de-identified secured data collection sheet.

### Statistical analysis

Continuous data was presented as mean ± standard deviation (SD) or median and IQR, whereas categorical data was presented as counts and percentages. Univariate analysis using Chi square test or non-parametric Wilcoxon rank test as appropriate was conducted to identify factors associated with ICU and hospital mortality. Factors included in the univariate analysis were: age, gender, APACHE II score, type of malignancy, chemotherapy received within 1 month of ICU admission, SEER stage, smoking history, neutropenia and thrombocytopenia upon ICU admission, acute kidney injury and elevated total bilirubin and lactic acid levels upon ICU admission, need for mechanical ventilation and having positive cultures. Multivariate logistic regression was performed for factors that were significant in the univariate analysis. Statistical significance was considered as a *p*-value ≤0.05. All analyses were performed using SAS version 9.4 (SAS Institute Inc., Cary, NC).

## Results

Over the study period, 1408 patients met the inclusion criteria. Patient demographics and baseline characteristics are outlined in Table 1. The mean age was 56.8 ± 16.1 (SD) years and mean APACHE II score was 23.0 ± 7.91 (SD). The majority of the included patients had solid malignancies (67.8%) and 402 (28.6%) received chemotherapy within 1 month prior to the ICU admission. Neutropenia and thrombocytopenia were reported in 19.3 and 39.5% of the patients, respectively, and mechanical ventilation was required for 42% of the patients. Acute kidney injury was reported upon ICU admission in 579 (41.1%) of the patients. Elevated total bilirubin level was reported in 195 (13.8%) and elevated lactic acid level was reported in 918 (65.2%) patients.

**Table 1:** Baseline characteristics and univariate analysis

Baseline characteristic	Value (***N*** = 1408)	ICU mortality		P-value	Hospital mortality		***P***-value
		Alive	Dead		Alive	Dead	
**Age (years), mean (SD)**	56.8 (16.1)	57.3	56.3	0.1770	56.3	57.1	0.4132
**Apache II Score, mean (SD)**	23.0 (7.91)	21.3	24.9	0.0000	20.4	24.5	0.0000
**Male gender, N (%)**	821 (58.3%)	437 (60.7%)	384 (55.8%)	0.06336	317 (64.2%)	504 (55.1%)	0.00104
**Type of malignancy**				0.7762			0.63671
**Hematology, N (%)**	**453 (32.2%)**	229 (31.8%)	224 (32.6%)		163 (33.0%)	290 (31.8%)	
Lymphoma	191 (13.6%)						
Leukemia	188 (13.4%)						
Multiple Myeloma	66 (4.6%)						
Others	8 (0.6%)						
**Solid, N (%)**	**955 (67.8%)**	491 (68.2%)	464 67.4%)		331 (67.0%)	624 (68.2%)	
Gastrointestinal Tumors	248 (17.6%)						
Breast Cancer	143 (10.2%)						
Lung Cancer	138 (9.8%)						
Gynecologic Tumors	69 (4.9%)						
Bladder Cancer	60 (4.3%)						
Prostate Cancer	43 (3.0%)						
Pancreatic Cancer	34 (2.4%)						
Renal Cell Carcinoma	31 (2.2%)						
Others	176 (12.5%)						
Unknown primary site	13 (0.9%)						
**SEER stage, N (%)**				< 0.0001			< 0.0001
In Situ	9 (0.6%)	5 (0.7%)	4 (0.6%)		3 (0.6%)	6 (0.7%)	
Localized	111 (7.9%)	74 (10.3%)	37 (5.3%)		55 (11.1%)	56 (6.1%)	
Regional	275 (19.5%)	170 (23.6%)	105 (15.3%)		124 (25.1%)	151 (16.5%)	
Distant	458 (32.5%)	193 (26.8%)	265 (38.5%)		123 (25%)	335 (36.7%)	
Unknown (Unstaged)	38 (2.7%)	16 (2.2%)	22 (3.2%)		6 (1.2%)	32 (3.5%)	
Not applicable	453 (32.2%)	229 (31.8%)	224 (32.6%)		163 (33.0%)	290 (31.7%)	
Not available	64 (4.5%)	33 (4.6%)	31 (4.5%)		20 (4%)	44 (4.8%)	
**Recent Chemotherapy, N (%)**				0.921			0.19771
Yes	402 (28.6%)	206 (28.6%)	196 (28.5%)		151 (30.7%)	251 (27.5%)	
No	999 (71%)	509 (71.2%)	490 (71.2%)		339 (68.6%)	660 (72.2%)	
No available data	7 (0.4%)	5 (0.2%)	2 (0.3%)		4 (0.7%)	3 (0.3%)	
**Smoking, N (%)**				0.37882			0.0191
Yes	631 (44.8%)	330 (45.8%)	301 (43.8%)		242 (49%)	389 (42.6%)	
Not available	117 (8.3%)	61 (8.5%)	56 (8.1%)		40 (8.1%)	77 (8.4%)	
**Neutropenia, N (%)**	272 (19.3%)	142 (19.7%)	130 (18.9%)	0.69443	106 (21.5%)	166 (18.2%)	0.13496
**Thrombocytopenia, N (%)**	556 (39.5%)	244 (33.9%)	312 (45.3%)	0.00001	157 (31.8%)	399 (43.7%)	0.00001
**Mechanical ventilation, N (%)**	591 (42%)	230 (31.9%)	361 (52.5%)	< 0.0001	138 (27.9%)	453 (49.6%)	< 0.0001
**Positive cultures, N (%)**	836 (59.4%)	380 (52.8%)	456 (66.3%)	< 0.0001	247 (50%)	589 (64.4%)	< 0.0001
**Acute kidney injury, N (%)**				0.37852			0.38032
Yes	579 (41.1%)	323 (44.9%)	256 (37.2%)		228 (46.1%)	351 (38.4%)	
No	551 (39.1%)	293 (40.1%)	258 (37.5%)		203 (41.1%)	348 (38.1%)	
Not available	278 (19.8%)	104 (15%)	174 (25.3%)		63 (12.8%)	215 (23.5%)	
**Elevated total bilirubin level mg/dl, N (%)**				0.00009			0.00004
Yes	195 (13.8%)	80 (11.1%)	115 (16.7%)		49 (9.9%)	146 (16%)	
No	875 (62.2%)	494 (68.6%)	381 (55.4%)		359 (72.7%)	516 (56.5%)	
Not available	338 (24%)	146 (20.3%)	192 (27.9%)		86 (17.4%)	252 (27.5%)	
**Elevated lactic acid level mmol/l, N (%)**				0.00164			0.00017
Yes	918 (65.2%)	437 (60.7%)	481 (69.9%)		284 (57.5%)	634 (69.4%)	
No	370 (26.3%)	212 (29.4%)	158 (23%)		155 (31.4%)	215 (23.5%)	
Not available	120 (8.5%)	71 (9.9%)	49 (7.1%)		55 (11.1%)	65 (7.1%)	

Positive cultures were reported in 836 (59.4%) patients, most commonly blood and respiratory, with approximately two-third of the cultures being Gram-negative pathogens (Table 2). The median ICU length of stay was 4 days (IQR 3–8) and the ICU and hospital mortality were reported in 688 (48.9%) and 914 (64.9%) patients, respectively.

**Table 2:** Type of cultures and microorganisms

Type of positive culture/microorganism	N (%)
Type of positive culture*, N	**1116 (100%)**
Blood culture	368 (33%)
Trap culture	297 (26.6%)
Urine culture	179 (16.1%)
Wound culture	132 (11.8%)
Others	140 (12.5%)
**Type of microorganisms**^**a**^	
**Gram negative bacteria**	**768 (68.8%)**
*Escherichia coli*	269 (35%)
*Klebsiella pneumoniae*	129 (16.8%)
*Pseudomonas aeruginosa*	125 (16.3%)
Acinetobacter baumannii	110 (14.3%)
Others	135 (17.5%)
**Gram positive bacteria**	**421 (37.7%)**
*Staphylococcus aureus*	142 (33.7%)
Coagulase-negative staphylococci	72 (17.1%)
Enterococcus species	65 (15.4%)
Streptococcus species	31 (7.4%)
Corynebacterium species	15 (3.6%)
Others	96 (22.8%)
**Fungal**	**147 (13.1%)**
Candida species	130 (88.4%)
Aspergillus	17 (11.6%)

^a^Eight hundred thirty-six patients had microbiologically proven infection; with a total of 1116 reported positive culture as patients could have more than one culture. Also, patients could have had more than one pathogen type in each culture type

In the logistic regression analysis of factors that were significant in the univariate analysis (Table 1), mechanical ventilation, thrombocytopenia, elevated total bilirubin, elevated lactic acid levels, presence of positive cultures and Apache II score within the first 24 h of admission were significantly associated with both ICU and hospital mortality as presented in Table 3.

**Table 3:** Predictors significantly associated with mortality

Predictor	Odds Ratio	95% Confidence Limits		***P***-value
**ICU mortality**				
Mechanical ventilation	2.109	1.682	2.643	<.0001
Apache II score	1.054	1.039	1.069	<.0001
Thrombocytopenia	1.611	1.285	2.020	<.0001
Positive culture	1.632	1.301	2.048	<.0001
Elevated total bilirubin level	1.940	1.366	2.757	0.0002
Elevated lactic acid level	1.355	1.009	1.820	0.0436
**Hospital mortality**				
Mechanical ventilation	2.224	1.740	2.844	<.0001
Apache II score	1.063	1.046	1.080	<.0001
Thrombocytopenia	1.640	1.287	2.089	<.0001
Positive culture	1.652	1.305	2.090	<.0001
Elevated total bilirubin level	2.311	1.555	3.436	<.0001
Elevated lactic acid level	1.477	1.095	1.992	0.0106

## Discussion

In this report, we described the outcomes of a large cohort of patients with solid and hematological malignancies admitted to the ICU with septic shock. In addition, we evaluated predictors of mortality in this patient population. In our 12-year study, about two-third of the patients treated for septic shock died during their hospitalization. Though the mortality rate reported in this study may be comparable to the outcomes reported by others for cancer patients with septic shock [16, 19], the high mortality rate highlights the importance of identifying measures to improve the outcomes in cancer patients with septic shock as well as developing prediction models to help in the early identification of patients with poor prognosis. We identified several factors associated with hospital mortality, with mechanical ventilation and elevated bilirubin levels having the highest association. Other factors were the APACHE II, thrombocytopenia, elevated lactic acid levels, and the presence of positive cultures.

De Montmollin et al. [16] evaluated the outcomes of 218 critically ill cancer patients with septic shock of pulmonary origin. The study included patients with both solid and hematological malignancies, but unlike our study, the majority of their patients had hematological malignancies. Though the proportion of patients that required mechanical ventilation was higher than that reported in our study (78.4% vs 42%), they described similar hospital mortality (62.4%). However, De Montmollin et al. did not provide any mortality prediction scores (e.g., APACHE II) for their patients and therefore, it would be difficult to determine if the patients in both studies had similar severity of illnesses. In their study, hospital mortality was associated with age older than 60 years, time between first symptoms and ICU admission, mechanical ventilation, and coma.

Regazzoni et al. [19] evaluated the outcomes and predictors of mortality in a group of 73 patients with cancer and septic shock. Though more patients required mechanical ventilation, compared to our study (61.6% vs 42%), the mean APACHE II was lower (21.5 vs 23). ICU mortality was reported in 53.4% of the patients and the need for mechanical ventilation and liver dysfunction were independent predictors of mortality. However, the APACHE II score was not identified as a significant mortality predictor.

Several other studies evaluated septic shock in cancer patients, but they included patients with sepsis and septic shock, with the proportion of septic shock patients ranging between 25 and 60% of the study population [4, 7, 13–19, 21, 23, 24]. Lemiale et al. [23] recently published the characteristics and outcomes of a large cohort of cancer patients treated with sepsis and septic shock at 7 European ICUs over two decades (1994–2015). Among the enrolled patients, 56.8% received vasopressors, 49.3% required mechanical ventilation, and the majority had hematological malignancies. The study reported a day-30 mortality in 39.9% of the patients and identified mechanical ventilation and vasopressors use as independent factors associated with mortality.

Thrombocytopenia upon admission was associated with increased ICU and hospital mortality in this study. Few studies evaluated the impact of thrombocytopenia on the outcomes of critically ill patients. Williamson et al. [25] reported that thrombocytopenia upon admission was independently associated with mortality, with the greatest impact in patients with certain conditions, one of which was cancer. In addition, Burunsuzoğlu et al. [24] reported a higher ICU mortality rate in those who developed thrombocytopenia during ICU admission in comparison to those who did not (40.3% versus 17.5%).

Despite the assumption that neutropenia may impact the outcomes of patients with serious infections, recent data has suggested otherwise. In our study, neutropenia was not identified as a significant predictor of mortality. In a meta-analysis conducted by Bouteloup et al. [26], though the unadjusted mortality of neutropenic patients with cancer was 11% higher, compared to non-neutropenic patients, this effect was not significant when adjusted for severity of illness. Similar observations were also reported by Regazzoni et al. in which neutropenia did not impact the outcomes of cancer patients with septic shock [19].

Data regarding the impact of total bilirubin level on mortality is limited. In our study, the presence of elevated total bilirubin level was associated with both ICU and hospital mortality. Ñamendys-Silva et al. [14] also reported that total bilirubin level is one of the predictors for organ failure that is associated with ICU mortality. On the other hand, Regazzoni et al. [19] did not find a significant association between liver dysfunction and mortality.

Similar to what was reported earlier by Hawari et al. [3] in cancer patients, our study showed that elevated lactic acid level within 24 h of ICU admission with septic shock was a predictor for mortality. Data evaluating the association between elevated lactic acid and mortality varied. One study [27] reported lactic acid as an independent factor to predict mortality in cancer patients admitted with sepsis, while another study concluded that lactic acid alone is insufficient to predict poor outcomes in this patient population [28].

The association between renal failure and increased mortality in critically ill cancer patients was reported in several studies either in the form of severe renal impairment that necessitated renal replacement therapy [23, 29, 30] or acute kidney injury despite the severity level [17]. In our study; acute kidney injury was not identified as a significant predictor of mortality; this could be related to the criteria we used to define acute kidney injury and the inability to distinguish those who needed renal replacement therapy. Lemiale et al. [23] published a large cohort study of cancer patients treated with sepsis and septic shock in which renal replacement therapy, but not acute kidney injury, was associated with poor outcomes.

In this study, the presence of positive cultures was identified as a predictor associated with both ICU and hospital mortality. Sigakis et al. [31] evaluated the outcomes of culture-negative and culture-positive sepsis in a large cohort of patients from the intensive care units, emergency department, and general wards. After adjusting for severity of illness, the authors reported no difference in outcomes between the two groups. In a study conducted by Azoulay et al. [4], similar findings were reported in patients with cancer, in which positive cultures were not associated with mortality. We hypothesis that the differences in findings may be related to the type of infections and the time to initiating antibiotics, especially in patients with multi-drug resistant Gram-negative pathogens. We previously reported higher mortality in patients with multi-drug resistant *Acinetobacter baumannii* who had appropriate antimicrobial therapy (i.e., colistin) initiated after 24 h of onset of sepsis [32]. Given that Gram-negative infections were the most common infection in our patients, and that cancer patients are at risk of infections with multi-drug resistant pathogens, the timing for initiating appropriate antibiotics may have contributed to this finding. However, this will need to be further explored to future studies.

This study has several limitations which we would like to highlight. The first related to the retrospective nature of the study which may have limited out ability to extract some of the necessary findings for patients with septic shock, such certain patient-related and infection-related features. In addition, we did not evaluate the goals of care and code status of patients during their ICU stay, which are factors that may impact the mortality outcome of patients. Furthermore, though we evaluated lactic acid upon admission, we did evaluate all subsequent lactic acid measurements and their impact of mortality. However, in an earlier study, we evaluated the predictive value of lactate in cancer patients with septic shock [33]. In the study, though normalization of lactate and clearance at 24 h were predictors of hospital mortality, they were not found to be strong predictors. Another limitation was being single centered and therefore, further multi-center studies are necessary to evaluate the outcomes of septic shock in cancer patients.

## Conclusions

In a relatively large cohort of patients with solid and hematological malignancies admitted to the ICU with septic shock, about two-third of the patients died prior to hospital discharge. Mechanical ventilation, APACHE II, thrombocytopenia, positive cultures, and elevated bilirubin and lactic acid levels were significant predictors of mortality. Future studies should identify measures to improve the outcomes of this patient population and to develop predictive models to identify patients with cancer and septic shock who may have poor outcomes.

## Data Availability

The datasets used and/or analyzed during the current study are available from the corresponding author on reasonable request.

## Abbreviations

ICU: Intensive Care Unit
ANC: Absolute Neutrophils Count
APACHE II score: Acute Physiology and Chronic Health Evaluation II score
SEER: Surveillance Epidemiology and End Results
